# Prevalence of biofilm producing *Acinetobacter baumannii* clinical isolates: A systematic review and meta-analysis

**DOI:** 10.1371/journal.pone.0287211

**Published:** 2023-11-30

**Authors:** Alemu Gedefie, Ermiyas Alemayehu, Ousman Mohammed, Getachew Mesfin Bambo, Samuel Sahile Kebede, Berhanu Kebede

**Affiliations:** 1 Department of Medical Laboratory Sciences, College of Medicine and Health Sciences, Wollo University, Dessie, Ethiopia; 2 Department of Medical Laboratory Sciences, College of Health Sciences, Mizan-Tepi University, Mizan, Ethiopia; 3 Department of Biomedical Sciences, College of Medicine and Health Sciences, Samara University, Samara, Ethiopia; Nitte University, INDIA

## Abstract

**Background:**

*Acinetobacter baumannii*, the first human pathogen to be designated as a "red-alert" pathogen, is on the critical priority list of pathogens requiring new antibiotics. Biofilm-associated diseases are the most common infections caused by the antibiotic-resistant bacteria *A*. *baumannii*. Multidrug-resistant strains are more easily transmitted around the world due to *A*. *baumannii’s* ability to produce biofilms, which allows it to develop antibiotic resistance mechanisms and thrive in healthcare environments. As a result, *A*. *baumannii* infections are becoming increasingly common in hospital settings allover the world. As a result, a comprehensive systematic review and meta-analysis were carried out to determine the global prevalence of biofilm-producing *A*. *baumannii* clinical isolates.

**Methods:**

Articles were extensively searched in bibliographic databases and grey literatures using entry terms or phrases. Studies meeting eligibility criteria were extracted in MS Excel and exported into STATA version 12 software for statistical analysis. A random-effects model was used to compute the pooled prevalence of biofilm-producing *A*. *baumannii* clinical isolates. The heterogeneity was quantified by using the I^2^ value. Publication bias was assessed using a funnel plot and Egger’s test. Sensitivity analysis was done to assess the impact of a single study on pooled effect size.

**Result:**

Of the 862 studies identified, 26 studies consisted of 2123 *A*.*baumannii* clinical isolates of which 1456 were biofilm-producing. The pooled prevalence of biofilm-producing *A*.*baumannii* clinical isolates was 65.63% (95% CI = 56.70%-74.56%). There was substantial heterogeneity with an I^2^ value of 98.1%. Moreover, 41.34%, 33.57%, and 27.63% of isolates of strong, mild, and weak producers of biofilm. Higher prevalence was found in studies published after 2014 (66.31%); Western Pacific region (76.17%); and Asia (66.22%) followed by the African continent (57.29%).

**Conclusion:**

The pooled prevalence of biofilm-producing *A*. *baumannii* clinical isolates has risen alarmingly, posing a public health risk. This indicates the burden of biofilm-producing *A*. *baumannii* infections urges routine screening and appropriate treatment for better management of hospitalized patients, as well as effective controlling of the emergence of drug resistance. Furthermore, this finding is an alert call for the stakeholders to develop strong infection prevention and antibiotics stewardship programs for the prevention and control of biofilm-producing bacterial infections.

## 1. Background

*Acinetobacter baumannii* is an opportunistic and emerging global antibiotic-resistant Gram-negative bacterium that emerged as a clinically relevant pathogen causing biofilm-associated infections such as ventilator-associated pneumonia and catheter-related infection, both of which are resistant to antibiotic therapy as well as a wide range of nosocomial infection outbreaks, community-acquired infections, or war- and natural disaster-related infections [1]. *A*. *baumannii* is a “red-alert” human pathogen listed as the first pathogen on the critical priority list of pathogens for novel antibiotics. It is also an ESKAPE pathogen (*Enterococcus faecium*, *Staphylococcus aureus*, *Klebsiella pneumoniae*, *Acinetobacter baumannii*, *Pseudomonas aeruginosa*, *and Enterobacter species*) [1,2].

*A*. *baumannii* causes a multitude of infections such as bacteremia, pneumonia, urinary tract infection, meningitis, and wound infection. Severe hospital-acquired infections are the predominant infections, particularly in the ICU [3]. Evidence also showed that the mortality rate for *A*. *baumannii* infected ICU patients ranges from 45 to 60%, and it can reach over 80% when these organisms exhibit widespread drug resistance [4]. The therapeutic options available for the treatment of infections caused by multiple drug-resistant (MDR) *A*. *baumannii* strains are still insufficient [5,6]. Thus, public health is currently seriously endangered by the rapidly spreading multidrug resistance in *A*. *baumannii*. The situation has become increasingly extreme in recent years due to an ongoing rise in MDR, extensive drug-resistant (XDR), and pan-drug-resistant (PDR) *A*. *baumannii* nosocomial isolates, some of which are even resistant to tigecycline and colistin, the last-resort medications in therapeutic protocols [7].

*A*. *baumannii’s* recent fast emergence as a multidrug-resistant strain has seriously harmed public health. By adhering to various biotic and abiotic surfaces, such as vascular catheters, cerebrospinal fluid shunts, or foleys catheters, Acinetobacter can easily live and spread in the hospital environment because of its propensity to form biofilms [8]. Biofilm formation is a complex process and is regulated by a variety of factors such as biofilm-associated protein (Bap), synthesis and assembly of pili; Outer membrane protein A (OmpA); biofilm growth-associated repressor (BigR). There is a connection between biofilm development and antimicrobial resistance, as evidenced by the fact that several biofilm-associated genes influence antimicrobial susceptibility [9–12]. All of these elements work together to both directly and indirectly contribute to the growth of anti-microbial medication resistance and biofilm formation.

*A*. *baumannii* is the most prevalent opportunistic pathogen in clinical samples, and because it may colonize hospitals and acquire resistance, it causes nosocomial infections that are challenging to treat [13]. Production of biofilm on invasive devices is a potent protection mechanism that is beneficial for both bacterial protection and the exchange of resistance genes among the participating cells. Furthermore, biofilm formation also heightens pathogenicity, exceptional drug resistance, treatment complications, facilitates bacterial colonization and survival traits in *A*. *baumannii* in hospitals and medical equipment [14]. Thus, biofilm-producing *A*.*baumannii* isolates are resistant to most antimicrobials, however, few effective treatments exist which will worsen the spread of the bacterium in a hospital environment as a result of biofilm formation on surfaces and the expression of multidrug resistance [15].

Generally, infections due to *A*. *baumannii* can be associated with a multitude of clinical challenges. Thus, infection control practice initiatives for the prevention of healthcare-acquired infections have been implemented. Moreover, comprehensive data is quite important for evidence-based decisions both in the clinical and public health areas. However, epidemiological information is still scarce, especially on the emergence of challenging biofilm-producing clinical isolates. Therefore, to resolve serious health crises with high socio-economic costs, evidence-based practices are quite essential. Therefore, knowing the epidemiology is critical to taking further preventive measures, designing alternative mitigation activities, and reducing its further spread and complications. Thus, this systematic review and meta-analysis are aimed at synthetically analyzing the prevalence of biofilm-producing *A*. *baumannii* clinical isolates.

## 2. Methods

### 2.1. Design and protocol registration

This systematic review and meta-analysis was designed to estimate the global pooled prevalence of *A*. *baumannii*. The result was reported based on Preferred Reporting Items for Systematic Review and Meta-analysis Protocols (PRISMA-P) [16] (**See Supporting information)**. The review protocol was registered in the International Prospective Register of Systematic Review (PROSPERO) under registration number CRD42022344156

### 2.2. Data source and search strategy

All articles regarding *A*. *baumannii* infection had been retrieved via a scientific search of electronic databases which includes PubMed/crucial, web of science, research Gate, and Scopus from May 10 to May 30, 2022. In addition to accounting for the studies’ omission during electronic database searches, a direct Google search was carried out using listed references in included articles.

The comprehensive and extensive searching strategy has been employed using condition, context, population, and outcome of interest (CoCoPop) formulating questions and searching terms were (“global”), (“worldwide”) and (‘‘prevalence”), (“epidemiology”) (“magnitude”), and (“biofilm”), (“*A*. *baumannii”)*, (“*Acinetobacter baumannii”)* and (“*Acinetobacter* species”). The search terms were combined using the Boolean operators "OR" and "AND" to fit the advanced searching of articles.

### 2.3. Study selection and quality assessment for risk of bias

Three independent authors (AG, EA, GM) identified the articles from databases and other sources. Duplicates were removed and four independent reviewers continued to screen the title and abstract of all potentially eligible studies. Then, the full text of potentially eligible studies that reported the prevalence or epidemiology of biofilm-producing *A*.*baumannii* infection was added to the collections for extraction. Disagreements between two independent reviewers (AG and EA) were settled by GM to reach a consensus. The quality of the articles was carefully assessed by three authors (AG, EA, GM). The full texts of the articles were used to determine whether the study met the selection criteria or whether the eligibility of the article was in doubt. The Joanna Brigg Institute (JBI) quality assessment manual was used to assess the methodological validity of each study design [17]. Using the critical appraisal checklists, studies were reviewed and articles with an average score of 50% to 75% were considered as good quality while greater than 75% score was defined as high quality. Thus, articles with both good and high quality were included in this systematic review and meta-analysis. Moreover, study qualities were assessed using the Newcastle-Ottawa Quality scale for cross-sectional studies [18].

### 2.4. Eligibility criteria

Original articles that reported the biofilm-producing *A*.*baumannii* clinical isolates across the globe were included. Studies reported only in English were included. On the other hand, studies reporting the biofilm-producing *A*.*baumannii* infection among environmental isolates were excluded. Furthermore, review articles, case reports, and letters to the editor were also excluded.

### 2.5. Outcome variables

The outcome variable for this study is the global pooled prevalence of biofilm-producing *A*.*baumannii* clinical isolates.

### 2.6. Data extraction

Data from the eligible studies were extracted by four reviewers (AG, EA, GM, and SS) independently in Microsoft Excel sheets. The information extracted from each study includes the name of the first author, publication year, WHO region, continent, number of *A*. *baumannii* isolates, and diagnostic methods of biofilm detection.

### 2.7. Statistical analysis

The data extraction was done using a Microsoft Excel worksheet and the meta-analysis was done by using STATA version 12 with metan commands. The point estimate and 95% confidence interval of the prevalence of biofilm-producing *A*.*baumannii* clinical isolates for the studies fulfilling inclusion criteria were calculated. Due to the high heterogeneity reported, the national pooled prevalence of biofilm-producing *A*.*baumannii* clinical isolates was calculated using a random effect model. DerSimonian Laird method was used to estimate the between-study variance. The Cochrane’s Q test and I^2^ statistics which provide an estimate of the percentage of variability in effect estimates that is due to heterogeneity rather than chance alone were used to assess the heterogeneity [19]. A p-value of < 0.005 was used to declare significant heterogeneity. Therefore, a random effect model was used to adjust the observed variability. Publication bias was assessed by visual observation of the symmetry of the funnel plot and Egger’s test statistics [20,21]. Sensitivity analysis was done to assess the impact of a single study on the overall pooled effect size. Subgroup analysis for the primary outcome was performed by publication year, WHO region, continent, and biofilm detection method.

## 3. Result

### 3.1. Description and search results of included articles

**Fig 1:**
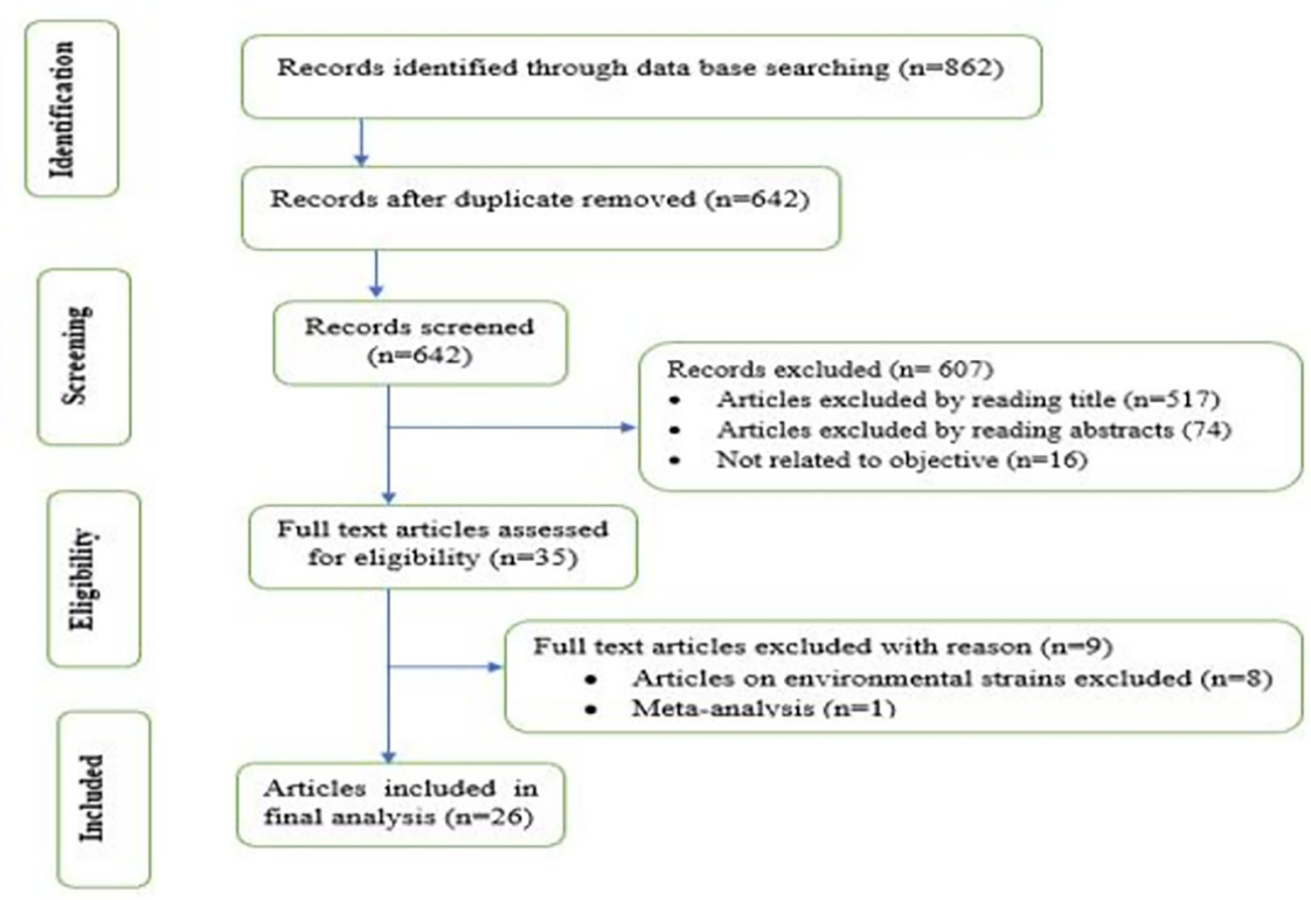
Flow diagram for the selection of eligible studies.

A total of 862 articles were retrieved from PubMed, Cochrane library, google scholar, Scopus, web of science, psych info, and African online archives. Six hundred and forty-two articles remained after removing 220 duplicate articles. From the remaining, 607 articles were excluded after reviewing the title, abstract, and objective of the study. Finally, 35 full-length articles were thoroughly reviewed by predetermined eligibility criteria, and 26 studies were found to be eligible for inclusion in this systematic review and meta-analysis. The preferred reporting items for systematic review and meta-analysis (PRISMA checklist 2009) were followed (Fig 1: Flow diagram for the selection of eligible studies) [16]. A total of 26 globally published articles with 2,123 *A*.*baumannii* clinical isolates were included in qualitative and quantitative meta-analyses, respectively. Of the total clinical isolates of *A*.*baumannii*, 1,456 were biofilm-producing isolates. The number of *A*.*baumannii* clinical isolates reported in studies ranged from 10 to 272 while biofilm-producing *A*.*baumannii* clinical isolates ranged from 4 to 249. The studies were conducted on three continents such as Asia, Africa, and South America. Of the 26 studies included in this systematic review and meta-analysis, 21 studies were conducted in Asian countries. Twenty-three studies were conducted from 2015 to 2021 while the rest were conducted from 2008 to 2014. Based on the WHO region, 10 studies were done in Eastern Mediterranean countries followed by 7 studies in South- Asian countries (Table 1).

**Table 1:** Characteristics of the included studies.

S.No	Author/ Reference	Publication year	Continent	Country	WHO Region	# *A*.*baumannii* isolate	# Biofilm cases	Prevalence of biofilm	Degree of biofilm		
									Strong; N(%)	Moderate; N(%)	Weak; N(%)
1	Asaad et al [22]	2021	Africa	Egypt	Eastern Mediterranean	94	66	70.21	19(28.8)	32(48.5)	15(22.7)
2	Khalil et al [7]	2021	Africa	Egypt	Eastern Mediterranean	165	54	32.73	33(61.1)	15(27.8)	6(11.1)
3	Madaha et al [23]	2020	Africa	Cameroon	Africa	27	19	70.37	7(36.8)	5(26.3)	7(36.8)
4	AL-Mousawi et al [24]	2018	Asia	Baghdad	Eastern Mediterranean	83	74	89.16	48(64.9)	16(21.6)	10(13.5)
5	Nesa et al [25]	2018	Asia	Bangladesh	South-east Asia	108	67	62.04	NT	NT	NT
6	Castilho et al [26]	2017	South America	Brazil	America	84	43	51.19	NT	NT	NT
7	DA SILVA et al [27]	2021	South America	Brazil	America	38	20	52.63	4(20.0)	11(55.0)	5(25.0)
8	Sung et al [28]	2018	Asia	Korea	Western Pacific	58	46	79.31	11(23.9)	32(69.6)	3(6.5)
9	Ryu et al [29]	2015	Asia	Korea	Western Pacific	49	24	48.98	NT	NT	NT
10	Li et al [9]	2021	Asia	China	Western Pacific	104	103	99.04	NT	NT	NT
11	Qi et al [30]	2016	Asia	China	Western Pacific	272	249	91.54	NT	NT	NT
12	Chen et al [31]	2020	Asia	China	Western Pacific	92	50	54.35	18(36.0)	17(34.0)	15(30.0)
13	Kumari et al [32]	2013	Asia	India	South-east Asia	65	45	69.23	7(15.6)	18(40.0)	20(44.4)
14	Rao et al [33]	2008	Asia	India	South-east Asia	55	34	61.82	NT	NT	NT
15	Asati et al [34]	2017	Asia	India	South-east Asia	10	4	40.00	NT	NT	NT
16	Badave et al [35]	2015	Asia	India	South-east Asia	72	45	62.50	37(82.2)	5(11.1)	3(6.7)
17	Gurung et al [36]	2014	Asia	India	South-east Asia	60	30	50.00	NT	NT	NT
18	Azizi et al [37]	2016	Asia	Iran	Eastern Mediterranean	65	54	83.08	23(42.6)	18(33.3)	13(24.1)
19	Dehbalaei et al [38]	2017	Asia	Iran	Eastern Mediterranean	48	35	72.92	3(8.6)	4(11.4)	28(80.0)
20	Moghadam et al [39]	2018	Asia	Iran	Eastern Mediterranean	64	31	48.44	NT	NT	NT
21	Babapour et al [40]	2016	Asia	Iran	Eastern Mediterranean	156	153	98.08	13(8.5)	91(59.5)	49(32.0)
22	Monfared et al [41]	2019	Asia	Iran	Eastern Mediterranean	118	86	72.88	16(18.6)	37(43.0)	33(38.4)
23	HASSAN et al [42]	2019	Asia	Iraq	Eastern Mediterranean	74	8	10.81	7(87.5)	1(12.5)	0(0.0)
24	Dumaru et al [43]	2019	Asia	Nepal	South-east Asia	63	34	53.97	NT	NT	NT
25	O¨ zkul et al [44]	2020	Asia	Turkey	Europe	44	41	93.18	36(87.8)	5(12.2)	0(0.0)
26	Elbehiry et al [45]	2020	Asia	Saudi Arabia	Eastern Mediterranean	55	41	74.55	18(43.9)	13(31.7)	10(24.4)

NT: Not Tested; N: Number; %: Percent.

### 3.2. Pooled prevalence of biofilm-producing *A*.*baumannii* clinical isolates

**Fig 2:**
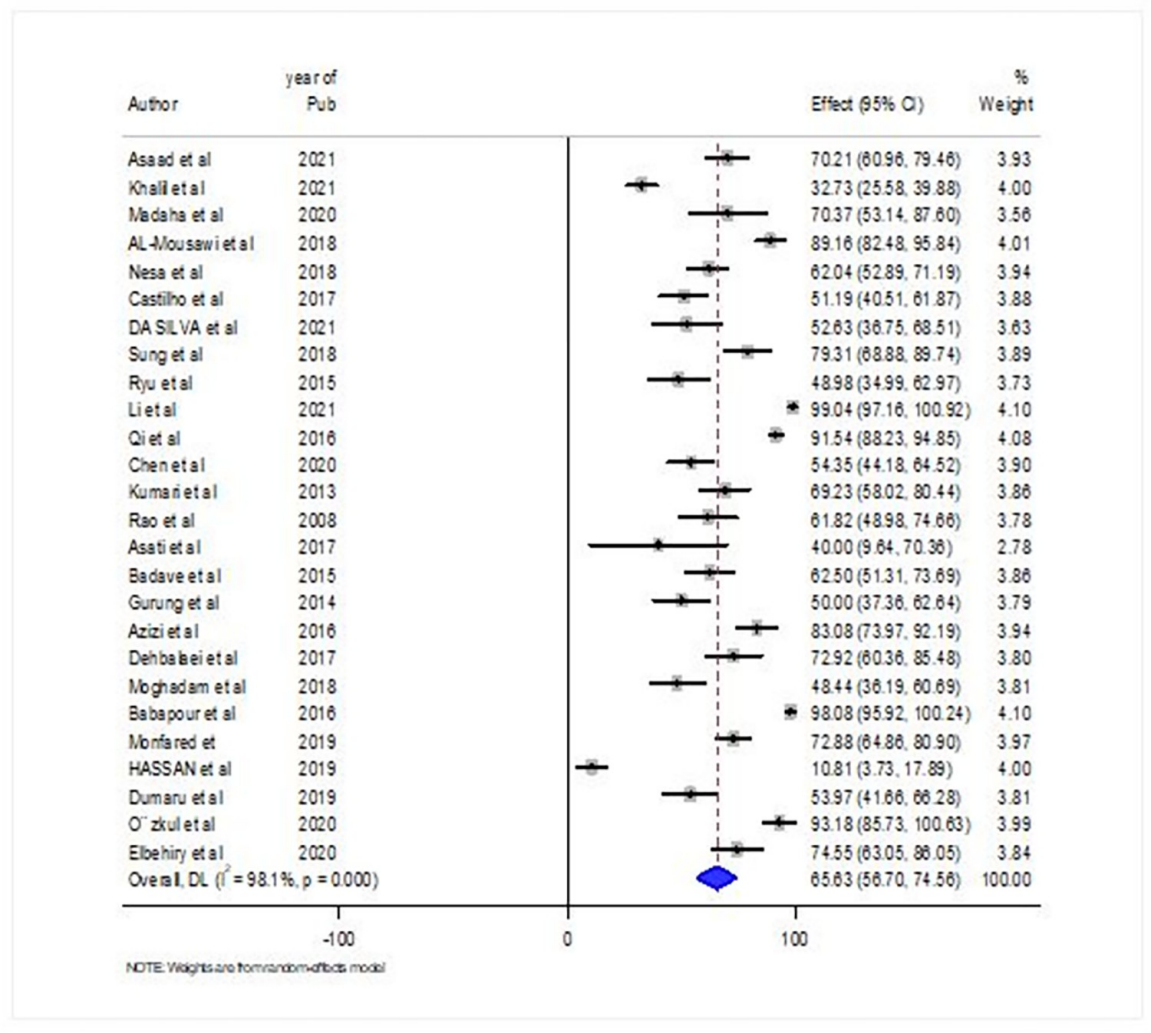
Forest plot showing the global prevalence of biofilm producing A.baumannii from2008-2021.

In this systematic review and meta-analysis, the global prevalence of biofilm-producing *A*.*baumannii* clinical isolates was 65.63% (95% CI = 56.70%-74.56%). Overall, the prevalence of biofilm-producing *A*.*baumannii* among the global population was variable, ranging from 10.81% reported from Iraq to 99.04% reported from China. There was substantial heterogeneity with an I^2^ of 98.1% (Fig 2: Forest plot showing the global prevalence of biofilm producing A.baumannii from2008-2021).

**Fig 3:**
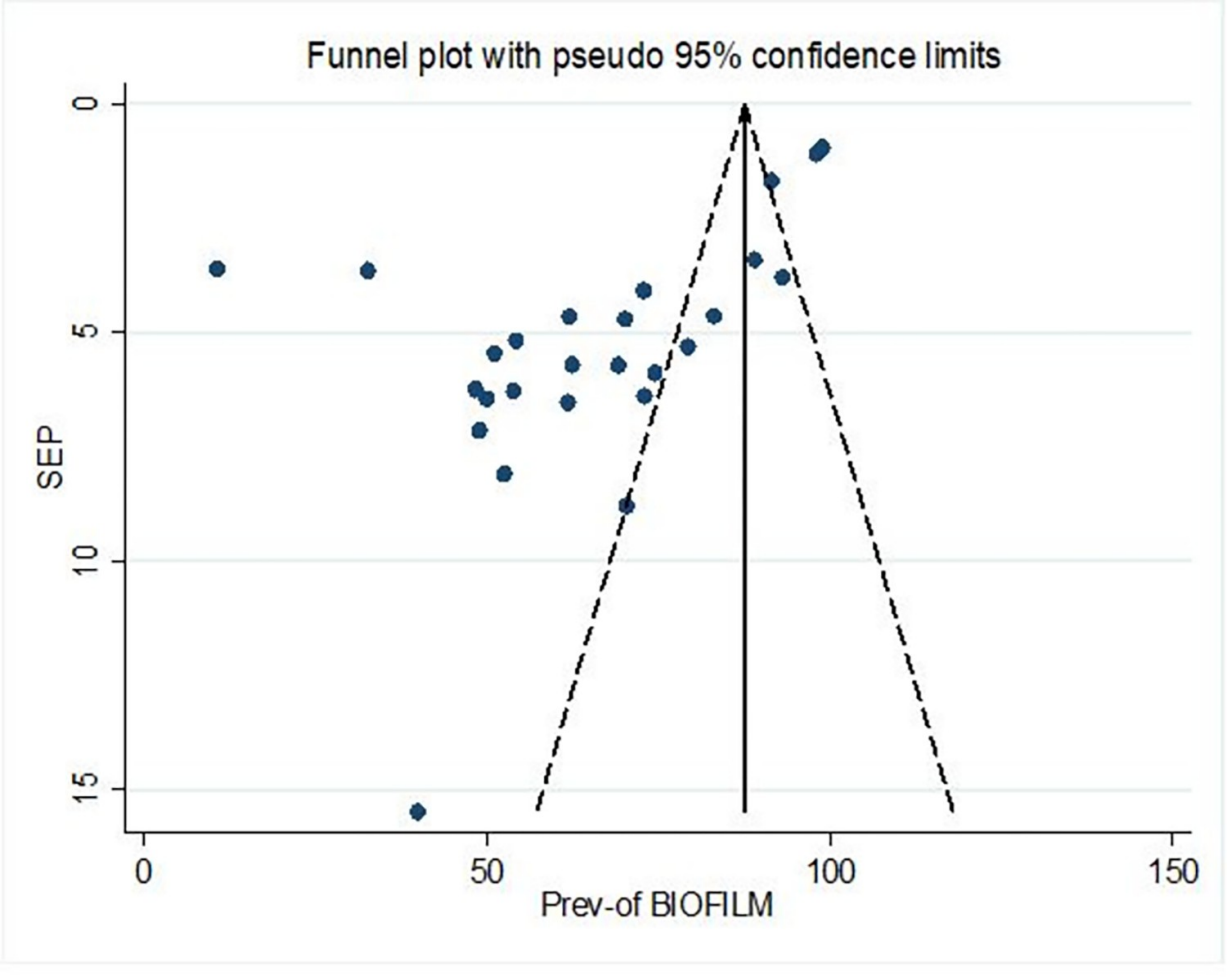
Funnel plot showing publication bias.

Subgroup analysis was done by publication year, continent, and WHO region. The global pooled prevalence rate of biofilm formation from 2015–2021 was 66.31% (95% CI: 56.86–75.75) as indicated in Fig 3. There was high heterogeneity with I^2^ of 98.2% among studies conducted between 2015 &2021 and there was no significant heterogeneity between the group (P = 0.446) (Table 2).

**Table 2:** Subgroup analysis by different categories of studies included in the systematic review and meta-analysis.

Subgroup	Category	Number of studies	Prevalence (95% CI)	P-value	I^2^	Heterogeneity between groups (P-value)
Publication year	2008–2014	3	60.63(49.48–71.77)	0.082	60.0%	0.446
	2015–2021	23	66.31(56.86–75.75)	**<0.0001**	98.2%	
Continent	Africa	3	57.29(28.89–85.70)	**<0.0001**	95.6%	0.037
	Asia	21	68.22(59.18–77.25)	**<0.0001**	97.9%	
	South America	2	51.64(42.78–60.50)	**<0.0001**	98.1%	
WHO region	Eastern Mediterranean	10	65.31(44.54–86.07)	**<0.0001**	98.9%	0.020
	Southeast Asia	7	59.77(54.34–65.19)	0.234	25.5%	
	America	2	51.64(42.78–60.50)	0.883	0.0%	
	Western Pacific	5	76.17(63.40–88.95)	**<0.020**	97.0%	
Biofilm detection method	Microtiter plate technique	14	70.42 (60.99–79.85)	**<0.0001**	97.8%	0.350
	Congo Red Agar	3	51.31(-1.61–104.23)	**<0.0001**	99.2%	
	Tissue culture plate	2	55.97(36.67–75.27)	0.173	46.1%	
Degree of biofilm	Strong	16	41.34(26.99–55.70)	**<0.0001**	96.5%	0.278
	Moderate	16	33.57(23.97–43.18)	**<0.0001**	90.2%	
	Weak	14	27.63(18.34–36.93)	**<0.0001**	91.3%	

Based on the existing available evidence, the pooled prevalence of biofilm-producing *A*.*baumannii* infection based on the continent of the studies showed that higher prevalence was found in Asia followed by Africa and the least was South America. The pooled prevalence of biofilm producing *A*.*baumannii* infection in Asia and Africa was 66.22% (95% CI:59.18–77.25) and 57.29% (95%CI:28.89–85.70), respectively. There was high heterogeneity among Asian studies and African studies with I^2^ of 97.9% (P = 0.000) and 95.6% (P = 0.000), respectively. However, there was no heterogeneity among South American studies with I^2^ of 0.0% (P = 0.883) as indicated in Table 2. Moreover, there was significant overall heterogeneity between subgroups i.e between continents (P = 0.0037) (Table 2).

Another subgroup analysis was done by the WHO region of studies. Studies from the Eastern Mediterranean region, Southeast Asian region, American and Western Pacific region were used for subgroup analysis. The higher prevalence (76.17%) of biofilm-producing *A*.*baumannii* infection was found in the Western Pacific region and the least prevalence (51.64%) was found in the American region. There was high heterogeneity among Western Pacific and Eastern Mediterranean regions with I^2^ of 97% and 98.9%, respectively. There was no heterogeneity among studies of the American region with I^2^ of 0.0% (P = 0.883). Moreover, there was significant overall heterogeneity between subgroups i.e between regions of WHO (P = 0.020) (Table 2).

Based on the laboratory method used for the detection of biofilm, studies done by the microtiter plate technique revealed 70.42% (95% CI = 60.99–79.85, P<0.0001) pooled prevalence of biofilm-producing *A*.*baumannii* with a substantially high level of heterogeneity with I^2^ of 97.8% (P <0.001). Moreover, there was no significant overall heterogeneity between subgroups i.e between types of biofilm detection methods (P = 0.073). Regarding the degree of biofilm; 41.34% (95% CI = 26.99–55.70, P<0.0001), 33.57% (95% CI = 23.97–43.18, P<0.0001), 27.63% (95% CI = 18.34–36.93, P<0.0001) of isolates of *A*.*baumannii* were strong, mild, and weak producers of biofilm. There was a higher heterogeneity of subgroups among the degree of biofilm despite there was no existing heterogeneity between groups (P = 0.278) (Table 2).

### 3.3. Sensitivity analysis

Each study did not affect the pooled estimate of the proportion indicating the precise aggregate result. When individual studies were omitted, the pooled effect size lay within the 95% confidence interval of the overall pooled effect size. This confirmed the absence of a single study impact on the overall pooled prevalence of biofilm-producing *A*.*baumannii* infection (Table 3).

**Table 3:** Sensitivity analysis of the included studies.

S.No	Author	Estimate	95% Confidence interval	
			Lower	Upper
1	Asaad et al	65.437157	56.279953	74.59436
2	Khalil et al	67.086639	58.656231	75.517044
3	Madaha et al	65.45282	56.345612	74.560028
4	AL-Mousawi et al	64.628929	55.334221	73.923637
5	Nesa et al	65.777405	56.675747	74.879074
6	Castilho et al	66.220901	57.19849	75.243317
7	DA SILVA et al	66.122749	57.058411	75.18708
8	Sung et al	65.069298	55.895138	74.243454
9	Ryu et al	66.280357	57.239441	75.321274
10	Li et al	64.125801	53.959618	74.291985
11	Qi et al	64.467644	54.615944	74.319351
12	Chen et al	66.093834	57.050842	75.136826
13	Kumari et al	65.482071	56.346775	74.617371
14	Rao et al	65.779716	56.680782	74.878654
15	Asati et al	66.364761	57.331387	75.39814
16	Badave et al	65.755234	56.651066	74.859406
17	Gurung et al	66.252548	57.216129	75288971
18	Azizi et al	64.904884	55.700352	74.109413
19	Dehbalaei et al	65.338203	56.201038	74.475372
20	Moghadam et al	66.318954	57.297016	75.340897
21	Babapour et al	64.157257	53.835232	74.479286
22	Monfared et	65.321342	56.129021	74.513672
23	HASSAN et al	68.170616	60.67728	75.663948
24	Dumaru et al	66.095787	57.037434	75.154144
25	O¨ zkul et al	64.471115	55.219807	73.722427
26	Elbehiry et al	65.268288	56.118126	74.418457
Combined*		65.629682	56.702953	74.556411

### 3.4. Publication bias

The funnel plot was used to assess the impact of the small-studies effect or publication bias on estimated pooled prevalence. The graph of the funnel plot becomes asymmetrical indicating the presence of publication bias (Fig 3: Funnel plot showing publication bias).

Furthermore, Egger’s test statistics confirmed the presence of marginally significant publication bias at a P-value < 0.001 (Table 4).

**Table 4:** Egger’s test statistics.

Standard Effect	Coefficient	Standard Error	T	P>|t|	95% Confidence Interval
Slope	104.832	4.306301	24.34	0.000	95.94424, 113.7198
Bias	-7.891943	1.495672	-5.28	0.000	-10.97886, -4.805027

### 3.5. Trim and fill analysis

Trim and fill analysis for each was conducted however trimming was not performed which will bring the data to be changed and the total number of studies remained at 26. The pooled proportion of biofilm producing *A*.*baumannii* clinical isolates was 65.63% at p-value < 0.001 (Hence, trim and fill analysis was performed (Table 5).

**Table 5:** Trim and fill analysis of the pooled proportion of biofilm-producing *A*.*baumannii* clinical isolates.

Meta-analysis Method	Pooled estimate	95% CI		Asymptotic		No. of Studies
		Lower	Upper	Z-value	P-value	
Fixed	87.609	86.503	88.716	155.156	<0.001	26
Random	65.630	56.703	74.556	14.410	<0.001	
Test for heterogeneity: Q = 1282.986 on 25 degrees of freedom (p < 0.001) Moment-based estimate of between studies variance = 505.168							
**Trimming estimator: Linear** **Meta-analysis type: Fixed-effect model**							
Iteration	Estimate	Tn	# to trim	Diff		
1	87.609	20	0	351		
2	87.609	20	0	0		
**Note**: No trimming performed, Data unchanged						
**Filled Meta-analysis**						
Meta analysis Method	Pooled estimate	95% CI		Asymptotic		No. of Studies
		Lower	Upper	Z-value	P-value	
Fixed	87.609	86.503	88.716	155.156	<0.001	26
Random	65.630	56.703	74.556	14.410	<0.001	

## 4. Discussion

In humans, an estimated 65% to 80% of microbial infections of all hospital infections are of biofilm origin. Once established, biofilm infections are very difficult to eradicate due to their resilience to removal by host defense mechanisms and antimicrobials [46]. This can be associated with the emergence of an alarming increment of multidrug-resistant strain that has seriously harmed public health. The present study revealed that the global pooled prevalence of biofilm-producing *A*.*baumannii* infection was 65.63% (95% CI = 56.70%-74.56%). The present comprehensive study finding showed that nearly seven in every ten *A*.*baumannii* infected patients had a probability of developing biofilm. Compared with the previous finding, the present finding was lower than the 90.5% pooled biofilm formation rate reported from burned patients [47]. This difference might be attributable to the target group where samples were taken. For example, burn victims are more susceptible to infections of all kinds due to the loss of their skin’s protective layer and the immunosuppression they undergo as a result of the systemic inflammatory response that the damaged tissue causes.

Evidences report revealed that biofilm-producing *A*.*baumannii* nosocomial isolates are so common. The survival, proliferation, and epidemic spread of the biofilm-producing *A*. *baumannii* in hospital settings, which results in resistance to many commercially available antibiotics and the expression of numerous virulence mechanisms, can be linked to the alarmingly increased prevalence of this organism [1,48]. This is because the biofilm encourages microbial adhesion and long-term survival on biotic and abiotic surfaces, which contributes to chronic and persistent infections, antimicrobial resistance, and strong survival in the hospital environment, particularly from immunocompromised patients in intensive care units [1,2]. Thus, biofilm-mediated infections, their resilience to therapy, and innovative treatment strategies become an emerging public health issue where novel approaches to treating these diseases are urgently needed because biofilm infections greatly increase patient morbidity and significantly increase healthcare expenses or costs [49]. Because biofilms are the primary source of the high frequency of *A*. *baumannii* infections linked to medical devices; infection control and treatment are extremely difficult [1,50]. This highlights the necessity of concentrating on infection prevention and control (IPC) measures or activities to manage *A*. *baumannii* biofilm-related illnesses caused by devices, as well as great selectivity in the application of treatments in conjunction with anti-biofilms such as octominin, a promising antibacterial and antibiofilm used for the control of multidrug-resistant *A*. *baumannii* [51].

Substantial heterogeneity with an I^2^ of 98.1% was found in the current study. The possible reasons for heterogeneity could be due to differences in methodological issues such as differences in the study design, the method of biofilm detection, target population types, variation in study settings like in facilities of developed and developing countries as well as periodic variation of studies. Moreover, it can be varied in association with infection prevention and control practices performances of health facilities aimed to be carried out to reduce hospital-acquired infection.

The global prevalence of biofilm-forming *A*. *baumannii* revealed that the Asian continent had the highest rate of infection (66.31%), which is consistent with earlier research suggesting that the treatment of infections brought on by MDR *A*. *baumannii* is challenging in Asian nations like Turkey, India, and Iran [52]. On the other hand, variations might be due to the target population involved in studies might be a population at risk who had a history of exposure to contaminated fomites, chronic pulmonary disease, receipt of broad-spectrum antibiotics & fluconazole, ICU admission and/or prolonged stay, mechanical ventilation and duration of mechanical ventilation, invasive procedures, devices, nasogastric tube, total parenteral nutrition [53–57]. In addition to this, variations in environmental conditions such as wet surfaces support the growth of biofilms.

The second high-burden continent next to Asia was found to be Africa where the prevalence was 57.29% which was higher than found in South America proving the need for routine screening of biofilm-producing *A*. *baumannii* infection. The discrepancy might be associated with variations in the developmental stages of the continent as well as the educational status of health care professionals and compliance level which in turn determines their quality of health care services. Furthermore, patients who are colonized or infected, antibiotic use selection pressure, and lax compliance with infection control protocols may all encourage persistence in hospital settings [54].

The present systematic review and meta-analysis showed that studies done by crystal violate staining method yields a higher prevalence (83.55%) of biofilm-producing *A*.*baumannii*. Moreover, our findings showed that 41.34% (95% CI = 26.99–55.70, P<0.0001), 33.57% (95% CI = 23.97–43.18, P<0.0001), 27.63% (95% CI = 18.34–36.93, P<0.0001) of isolates of *A*. *baumannii* were strong, moderate and weak biofilm producers which were consistent with 51.1%, 24.5%, and 25.8% biofilm former rates with their respective order reported from a previous systematic review and meta-analysis carried-out on burn patients (47)

The results of sensitivity analysis proved that there is no single study that impacted the pooled effect size. The pooled prevalence of biofilm-producing *A*. *baumannii* was calculated by excluding each study in turn, and the computed pooled prevalence was within 95% confidence intervals of the overall pooled prevalence.

There are some limitations to this study. First, the included studies were conducted only in 13 countries from Asia, Africa, and South America. There were no studies in Europe, the Middle East, Latin America, and other corners of the world. This might introduce a little bit of bias in justifying the intercontinental comparisons. Next, there was substantial heterogeneity observed between the studies, which may affect the interpretation of the results.

### 4.1. Conclusion

According to this systematic review and meta-analysis, the pooled prevalence of biofilm-producing *A*. *baumannii* clinical isolates has alarmingly increased and become a public health threat. This indicates the burden of biofilm-producing *A*. *baumannii* infections urges the need for routine screening and appropriate treatment for better management of hospitalized patients as well as effective controlling of the emergence of drug resistance. Furthermore, it serves as a wake-up call to international, continental, and national health bureaus, as well as other stakeholders, to develop targeted prevention and control strategies for nosocomial or hospital-acquired infections. This finding is also an alert call for the stakeholders to develop strong infection prevention and antibiotics stewardship programs for the prevention and control biofilm producing bacterial infections. Moreover, the data could be used for future complementary research and evidence-based decision-making both in clinical and public health approaches.

## Supporting information

S1 Checklist PRISMA 2020 checklist. (DOCX)

S1 File (XLSX)

S2 File (ZIP)

## Abbreviations

ICU: Intensive Care Unit
IPC: Infection Prevention and Control
MDR: Multiple Drug-Resistant
WHO: World Health Organization
