# Supplementary material for: Prevalence of biofilm producing Acinetobacter baumannii clinical isolates: A systematic review and meta-analysis
Source: PLoS One. 2023 Nov 30;18(11):e0287211. doi: 10.1371/journal.pone.0287211 (PMC10688650; doi:10.1371/journal.pone.0287211)
Supplement: S2 File — (ZIP) [file pone.0287211.s003.zip › Supplementary information 1.docx]

**Supplementary information-1**: Sensitivity analysis of the included studies

| **S.No** | **Author** | **Estimate** | **95% Confidence interval** | |
| --- | --- | --- | --- | --- |
|  |  |  | **Lower** | **Upper** |
| 1 | Asaad et al | 65.437157 | 56.279953 | 74.59436 |
| 2 | Khalil et al | 67.086639 | 58.656231 | 75.517044 |
| 3 | Madaha et al | 65.45282 | 56.345612 | 74.560028 |
| 4 | AL-Mousawi et al | 64.628929 | 55.334221 | 73.923637 |
| 5 | Nesa et al | 65.777405 | 56.675747 | 74.879074 |
| 6 | Castilho et al | 66.220901 | 57.19849 | 75.243317 |
| 7 | DA SILVA et al | 66.122749 | 57.058411 | 75.18708 |
| 8 | Sung et al | 65.069298 | 55.895138 | 74.243454 |
| 9 | Ryu et al | 66.280357 | 57.239441 | 75.321274 |
| 10 | Li et al | 64.125801 | 53.959618 | 74.291985 |
| 11 | Qi et al | 64.467644 | 54.615944 | 74.319351 |
| 12 | Chen et al | 66.093834 | 57.050842 | 75.136826 |
| 13 | Kumari et al | 65.482071 | 56.346775 | 74.617371 |
| 14 | Rao et al | 65.779716 | 56.680782 | 74.878654 |
| 15 | Asati et al | 66.364761 | 57.331387 | 75.39814 |
| 16 | Badave et al | 65.755234 | 56.651066 | 74.859406 |
| 17 | Gurung et al | 66.252548 | 57.216129 | 75288971 |
| 18 | Azizi et al | 64.904884 | 55.700352 | 74.109413 |
| 19 | Dehbalaei et al | 65.338203 | 56.201038 | 74.475372 |
| 20 | Moghadam et al | 66.318954 | 57.297016 | 75.340897 |
| 21 | Babapour et al | 64.157257 | 53.835232 | 74.479286 |
| 22 | Monfared et | 65.321342 | 56.129021 | 74.513672 |
| 23 | HASSAN et al | 68.170616 | 60.67728 | 75.663948 |
| 24 | Dumaru et al | 66.095787 | 57.037434 | 75.154144 |
| 25 | O¨ zkul et al | 64.471115 | 55.219807 | 73.722427 |
| 26 | Elbehiry et al | 65.268288 | 56.118126 | 74.418457 |
| **Combined*** | | 65.629682 | 56.702953 | 74.556411 |
