# Supplementary material for: Prevalence of biofilm producing Acinetobacter baumannii clinical isolates: A systematic review and meta-analysis
Source: PLoS One. 2023 Nov 30;18(11):e0287211. doi: 10.1371/journal.pone.0287211 (PMC10688650; doi:10.1371/journal.pone.0287211)
Supplement: S2 File — (ZIP) [file pone.0287211.s003.zip › Supplementary information 2.docx]

**Supplementary information-2**: Eggers test statistics

| Std-Eff | Coef. | Std.Err. | t | P | 95% Conf. Interval |
| --- | --- | --- | --- | --- | --- |
| Slope | 104.832 | 4.306301 | 24.34 | <0.001 | 95.94424, 113.7198 |
| Bias | -7.891943 | 1.495672 | -5.28 | <0.001 | -10.97886, -4.805027 |

Std Eff: Standard Effect; Coef: Coefficient: T- test Statistics; Std. Err: Standard Error; P: P-value of significance by assuming null zero value; Conf. Interval: Confidence Interval.
