# Supplementary material for: Prevalence of biofilm producing Acinetobacter baumannii clinical isolates: A systematic review and meta-analysis
Source: PLoS One. 2023 Nov 30;18(11):e0287211. doi: 10.1371/journal.pone.0287211 (PMC10688650; doi:10.1371/journal.pone.0287211)
Supplement: S2 File — (ZIP) [file pone.0287211.s003.zip › Supplementary information 3.docx]

**Supplementary information-3** Trim and fill analysis of pooled proportion of biofilm producing *A.baumanii* clinical isolates.

| Meta analysis Method | Pooled estimate | 95% CI | | Asymptotic | | No. of Studies |
| --- | --- | --- | --- | --- | --- | --- |
|  |  | Lower | Upper | Z-value | P-value |  |
| Fixed | 87.609 | 86.503 | 88.716 | 155.156 | <0.001 | 26 |
| Random | 65.630 | 56.703 | 74.556 | 14.410 | <0.001 |  |

Test for heterogeneity: Q= 1282.986 on 25 degrees of freedom (p < 0.001) Moment-based estimate of between studies variance = 505.168

| **Trimming estimator: Linear**  **Meta-analysis type: Fixed-effect model** | | | | | | | | | | |
| --- | --- | --- | --- | --- | --- | --- | --- | --- | --- | --- |
| Iteration | Estimate | | | Tn | | # to trim | | | Diff | |
| 1 | 87.609 | | | 20 | | 0 | | | 351 | |
| 2 | 87.609 | | | 20 | | 0 | | | 0 | |
| **Note**: No trimming performed, Data unchanged | | | | | | | | | | |
| **Filled Meta-analysis** | | | | | | | | | | |
| Meta analysis Method | | Pooled estimate | 95% CI | | | | Asymptotic | | | No. of Studies |
|  |  |  | Lower | | Upper | | Z-value | P-value | |  |
| Fixed | | 87.609 | 86.503 | | 88.716 | | 155.156 | <0.001 | | 26 |
| Random | | 65.630 | 56.703 | | 74.556 | | 14.410 | <0.001 | |  |
